# Prevalence of Type 2 Diabetes among Newly Detected Pulmonary Tuberculosis Patients in China: A Community Based Cohort Study

**DOI:** 10.1371/journal.pone.0082660

**Published:** 2013-12-18

**Authors:** Qiuzhen Wang, Aiguo Ma, Xiuxia Han, Shanliang Zhao, Jing Cai, Yunbo Ma, Jie Zhao, Yuwen Wang, Huaifeng Dong, Zhenlei Zhao, Lai Wei, Tao Yu, Peixue Chen, Evert G. Schouten, Frans J. Kok, Anil Kapur

**Affiliations:** 1 Nutrition Institute, Qingdao University, Qingdao, China; 2 Linyi Chest Hospital, Linyi, China; 3 Yishui Tuberculosis Clinic, Linyi, China; 4 Tancheng Tuberculosis Clinic, Linyi, China; 5 Yinan Tuberculosis Clinic, Linyi, China; 6 Lanshan Tuberculosis Clinic, Linyi, China; 7 Feixian Tuberculosis Clinic, Linyi, China; 8 Pingyi Tuberculosis Clinic, Linyi, China; 9 Cangshan Tuberculosis Clinic, Linyi, China; 10 Division of Human Nutrition, Wageningen University, Wageningen, The Netherlands; 11 World Diabetes Foundation, Gentofte, Denmark; Public Health Agency of Barcelona, Spain

## Abstract

**Background:**

Patients with type 2 diabetes (DM) have a higher risk of developing pulmonary tuberculosis (PTB); moreover, DM co-morbidity in PTB is associated with poor PTB treatment outcomes. Community based prevalence data on DM and prediabetes (pre-DM) among TB patients is lacking, particularly from the developing world. Therefore we conducted a prospective study to investigate the prevalence of DM and pre-DM and evaluated the risk factors for the presence of DM among newly detected PTB patients in rural areas of China.

**Methods and Findings:**

In a prospective community based study carried out from 2010 to 2012, a representative sample of 6382 newly detected PTB patients from 7 TB clinics in Linyi were tested for DM. A population of 6674 non-TB controls from the same community was similarly tested as well. The prevalence of DM in TB patients (6.3%) was higher than that in non-TB controls (4.7%, p<0.05). PTB patients had a higher odds of DM than non-TB controls (adjusted OR 3.17, 95% CI 1.14–8.84). The prevalence of DM increased with age and was significantly higher in TB patients in the age categories above 30 years (p<0.05). Among TB patients, those with normal weight (BMI 18.5–23.9) had the lowest prevalence of DM (5.8%). Increasing age, family history of DM, positive sputum smear, cavity on chest X-ray and higher yearly income (≥10000 RMB yuan) were positively associated and frequent outdoor activity was negatively associated with DM in PTB patients.

**Conclusions:**

The prevalence of DM in PTB patients was higher than in non-TB controls with a 3 fold higher adjusted odds ratio of having DM. Given the increasing DM prevalence and still high burden of TB in China, this association may represent a new public health challenge concerning the prevention and treatment of both diseases.

## Introduction

The association between diabetes mellitus (DM) and tuberculosis (TB) has been recognised for centuries. DM was a well-known risk factor for TB [1], but this association was nearly forgotten after the advent of widely available treatment for both diseases. With the current global increase in DM largely driven by increasing prevalence in the developing world, the DM population is anticipated to reach 552 million by 2030 [2] and the link is re-emerging. The co-morbidity of DM and TB represents a double burden with significant public health implications as recently recognised by several authors [3], [4]. In developing countries such as India, China, Bangladesh, Indonesia and Brazil, where TB is still highly endemic [5], the double burden and interaction of DM and TB will be more ominous. China accounts for nearly 17% of the world's TB burden, with an estimated 1.5 million new cases and approximately 270,000 deaths each year. In the meantime, the country has also witnessed an escalating epidemic of DM [2] as the consequence of industrialization, urbanization, increase in life expectancy, and changes in lifestyle in recent years. It was recently reported that the age standardised prevalence of DM and impaired glucose tolerance (IGT) reached to 9.7% and 15.5% respectively in China [6].

Jeon CY et al carried out a large meta analysis and discovered that DM patients were 3.1 times (95% CI 2.27–4.26) more likely to have TB than non-diabetic controls [7] and it has been estimated that the TB risk attributable to DM was between 15% and 25% [8], [9]. Two large scale longitudinal cohort studies from Korea and UK have shown similar findings with risk ratios of 3.47 (95% CI 2.98–4.03) and 3.80 (95% CI 2.30–6.10) [10], [11]. The mechanism behind the association between TB and DM is not fully understood but studies suggest that DM depresses the immune response through effects on macrophage and lymphocyte function, which in turn facilitates active TB disease. Conversely, it is also possible that TB can induce glucose intolerance and also deteriorate glycemic control in subjects with DM [12].

However, only a few studies have reported on screening for DM in TB patients. A wide range of DM prevalence among TB patients (1.9% to 39%) has been reported and most of the studies are based on secondary data analysis, self-reported DM, or have a small sample size [7], [10], [13]. Gaining a deeper understanding of the differences between TB patients with and without DM is urgently needed to prevent the co-morbidity and to improve the prognosis of the patients with DM and TB. Therefore, we initiated this large scale prospective epidemiologic study using primary data to identify the current prevalence of DM and pre-DM in newly-diagnosed PTB patients together with non-TB controls from the same community. Also, the odds ratios for DM among TB patients were analysed in order to get the clues to the early detection of DM in TB patients, and to figure out DM patient with what characteristics should be given priority to the prevention of TB.

## Methods

### Ethics Approval

This study was carried out in accordance with requirements documented in the Declaration of Helsinki. Ethics approval was obtained from the medical ethics committee of Qingdao Disease Prevention and Control Centre, Qingdao, People's Republic of China. All participants were fully informed and gave their written informed consent. This trial is registered in the Chinese Clinical Trial Registry (No. ChiCTR-OCC-10000994, URL: http://www.chictr.org/cn/proj/show.aspx?proj=411).

### Study Population

The study population was selected from Linyi rural area, Shandong province in North China. Seven TB clinics were randomly selected for this study including Yishui, Yinan, Lanshan, Cangshan, Tancheng, Feixian and Pingyi. Each TB clinic had a defined catchment area comprising approximately 0.9 million inhabitants. The diagnosis of PTB was made within the existing TB prevention and control system in China, in which clinical manifestations, sputum smear microscopy and chest radiography were the central component. Suspected PTB person was investigated by sputum smear examination. The patient was diagnosed as smear-positive PTB if sputum specimens were smear positive; if sputum smears were negative and chest radiograph was compatible with active PTB, the patient was diagnosed as smear-negative PTB after discussion by clinical and radiographic doctors [9]. All adult (≥18 years) newly-diagnosed PTB patients who registered for Directly Observed Treatment, Short Course (DOTS) in these TB clinics from September 2010 to December 2012 were included. HIV-positive patients were excluded because of the influence of antiretroviral therapy on insulin resistance as were subjects with type 1 diabetes.

A sample size of 7000 was calculated, assuming a prevalence of DM as 6.7% amongst the TB subjects as reported in the literature [14], considering non-response rate of 20% [15]. We adjusted it to 6200 because of better compliance of the participants observed in the pilot study than expected (modifying non-response rate to 10%). For estimating DM and pre-DM prevalences amongst the non-TB cohort, cluster random sampling was used to recruit subjects from the same communities as the TB cases.

Diagnosis of DM and pre-DM was based on WHO criteria for the classification of glucose tolerance based on fasting plasma glucose (FPG) [16]. After an overnight fast, venous blood of each participant was collected. Glucose oxidase method was used to estimate FPG level. Those with FPG≥6.1 mmol/L were referred to DM clinics for diagnostic confirmation with a second FPG test. Those with FPG level in the range of 6.1 mmol/L to 6.9 mmol/L were screened as pre-DM; those with FPG level ≥7.0 mmol/L were screened as DM. Lipid indexes including total cholesterol, triglyceride and HDLC were estimated by enzymatic procedure.

Anthropometric measurements including height and weight by standard procedure were measured by trained investigators. Body mass index (BMI, kg/m^2^) was calculated by using the formula: BMI = Weight (kg)/Height^2^ (m^2^). Underweight, normal weight, overweight, and obesity were defined by using the modified criteria for Chinese population [17]. The BMI cut-off value for underweight (severe underweight, moderate underweight, mild underweight), normal weight, overweight, and obesity was <18.5 kg/m^2^ (<16 kg/m^2^, 16∼16.9 kg/m^2^, 17∼18.4 kg/m^2^), 18.5∼23.9, 24.0∼27.9 kg/m^2^ and ≥28.0 kg/m^2^, respectively. Two blood pressure measurements were taken using sphygmomanometer with the subject in sitting posture, and the average of the two readings was recorded.

A structured questionnaire was administered to obtain information regarding socio-demographics, personal and family disease history, and lifestyle risk factors including smoking, alcohol consumption, educational level, outdoor activity, yearly income and marital status. The interviews were conducted by trained local TB workers in order to assure compliance and quality of the data collected.

### Data Management and Quality Control

Double data entry was carried out, and then a computer based error detection system was used to check the consistency of data. In case of inconsistency, the original questionnaire was checked and the error corrected via a re-entry. During the investigation, different methods of quality control including inter-lab comparison of glucose measurement were carried out.

### Statistical Analysis

The study design followed STROBE Guidelines [18]. SPSS version 19.0 was used for statistical analysis. Characteristics of TB and non-TB controls were compared and the study characteristic of TB patients by diabetes status including normoglycemia (Non-DM), prediabetes (Pre-DM), and diabetes (DM) were analyzed. Mean and standard deviation for continuous variables and proportions for categorical variables are reported. Independent sample t test and ANOVA were used to test continuous variables. Chi-square or Fisher's exact test was used to compare categorical variables. Kruskal-Wallis H test was used to compare ranked variable. Two multinomial logistic regression analyses were performed. The variables for inclusion in the multivariate model were chosen based on plausibility and variables with p values of <0.1 in univariate analysis were entered into the multivariate analysis. One multivariate logistic analysis was to examine the association between PTB and DM and pre-DM and to calculate odd ratios and 95% confidence intervals. The dependent variable was either DM or pre-DM, the independent variables being suffering from active PTB and we examined the following covariates for the effect modification or confounding: age [(years) (categorized in 4 units: <30, 30–39, 40–49, ≥50], sex, BMI [(kg/m2) (categorized as <18.5, 18.5–23.9, ≥24.0)]), yearly income [(RMB yuan) (categorized as<2000, 2000–9999, ≥10000], family history of DM, smoking, alcohol consumption, outdoor activity, education level and marital status. The other logistic regression analysis was performed to qualify the odds of having DM and pre-DM in active PTB patients, the dependent variable being either DM or pre-DM and the independent variables being the same as the first model without active TB and with PTB profile including positive sputum smear, cavity and involved lung field on chest radiograph. The entry probability was p≤0.05 and the removal probability was p>0.10. All variables were checked for collinearity in both models. The model fit was significant for both multinomial logistic regression analyses (χ^2^ = 285.6 and 372.8, respectively, p<0.001) and the fit was good (χ^2^ = 1093.47 and 1067.52, respectively, p>0.05). A p value of <0.05 was considered statistically significant.

## Results

**Figure 1:**
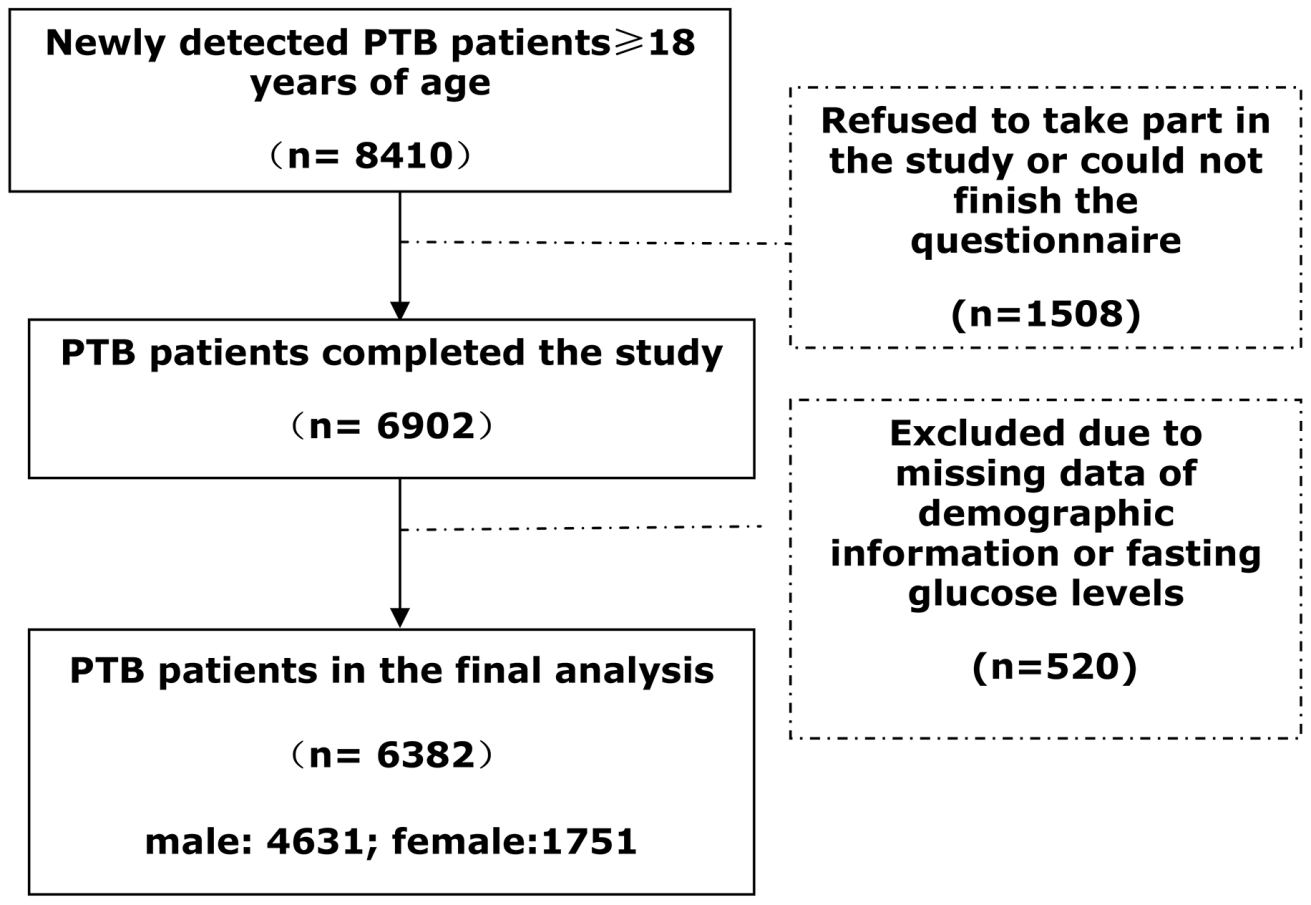
Flowchart of the pulmonary tuberculosis patients in the study, Linyi, 2010–2012. The details on the total number of eligible participants, those who have given written consent to undergo screening of DM and the patients included in the final analysis are illustrated in Figure 1. There were 8410 newly detected pulmonary tuberculosis patients in total study period. Finally, 6382 patients were included in the final analysis.

The details on the total number of eligible PTB patients, those who gave written consent to undergo screening of DM and those included in the final analysis are illustrated in a flow chart (Figure 1). A total of 8410 patients of ≥18 years of age were registered for TB treatment in the above 7 TB clinics in the study period. Finally, 6902 PTB patients completed the study. After the exclusion of 520 persons for whom demographic information or fasting glucose levels were missing, 6382 patients (4627 men and 1715 women) were included in the final analysis. The recorded response rate was 92.5%.

### General Characteristics

The demographic and anthropometric information of the TB and non-TB cohort is detailed in Table 1.

**Table 1:** Characteristics of TB patients and non-TB controls, Linyi, 2010–2012.

	TB(n = 6382)	Non-TB (n = 6675)	p value
**Male, n(%)**	4631(72.90)	3712(55.61)	<0.001
**Age**	50.41±18.63	50.77±16.40	0.242
**BMI**	20.91±2.76	22.52±2.99	<0.001
**Sbp**	119.69±11.43	123.95±15.00	<0.001
**Dbp**	76.70±7.70	79.31±9.80	<0.001
**Plasma glucose**	5.39±1.88	5.15±1.20	<0.001
**Hemoglobin**	131.89±17.25	131.07±43.48	0.610
**Total cholesterol**	4.39±1.09	4.69±1.20	<0.001
**HDLC**	1.46±0.60	1.63±0.82	<0.001
**Triglyceride**	1.20±1.11	1.31±0.94	0.884
**DM Family history, n (%)**	695(11.4)	765(12.6)	<0.001
**Smoking, n (%)**	900(14.1)	379(5.7)	<0.001
**Alcohol consumption, n (%)**	414(6.5)	753 (11.6)	<0.001
**Yearly income, n(%)**			
<2000	883(13.9)	935(14.0)	0.002
2000∼9999	3937(61.9)	4507(67.5)	
≥10000	1541(24.2)	1232(18.5)	
**Marital status, n(%)**			
Married	4694(79.4)	5419(87.8)	<0.001
Single/widowed/divorced	1221(20.6)	754(12.2)	
**Educational status, n(%)**			
illiteracy	1625(27.4)	1553(25.2)	<0.001
primary or middle school	3558(59.9)	3300(53.5)	
high school or higher	758(12.8)	1312(21.3)	
**Outdoor activity, n (%)**			
>2 hours/d	3779(63.6)	4041(67.5)	<0.001
≤2 hours/d	1863(31.4)	1644(27.5)	
None	300(5.0)	302(5.0)	

Data from 6382 TB patients and 6675 non-TB controls was available for analysis. The mean age of the PTB patients was 50.4±18.6 and of the non-TB controls 50.8±16.4 (p>0.05). There was a significant sex difference between TB and non-TB (p<0.001). Mean BMI of non-TB cohort was significantly higher than that of the TB cohort (p<0.001). Systolic blood pressure and diastolic blood pressure were higher in non-TB cohort compared to TB cohort (p<0.001). Plasma glucose level was higher in TB patients compared to non-TB controls (p<0.001). Smoking was more common in TB patients than in non-TB controls (p<0.001), while alcohol consumption was higher in non-TB (p<0.001).

### Relationship Between PTB and DM

**Figure 2:**
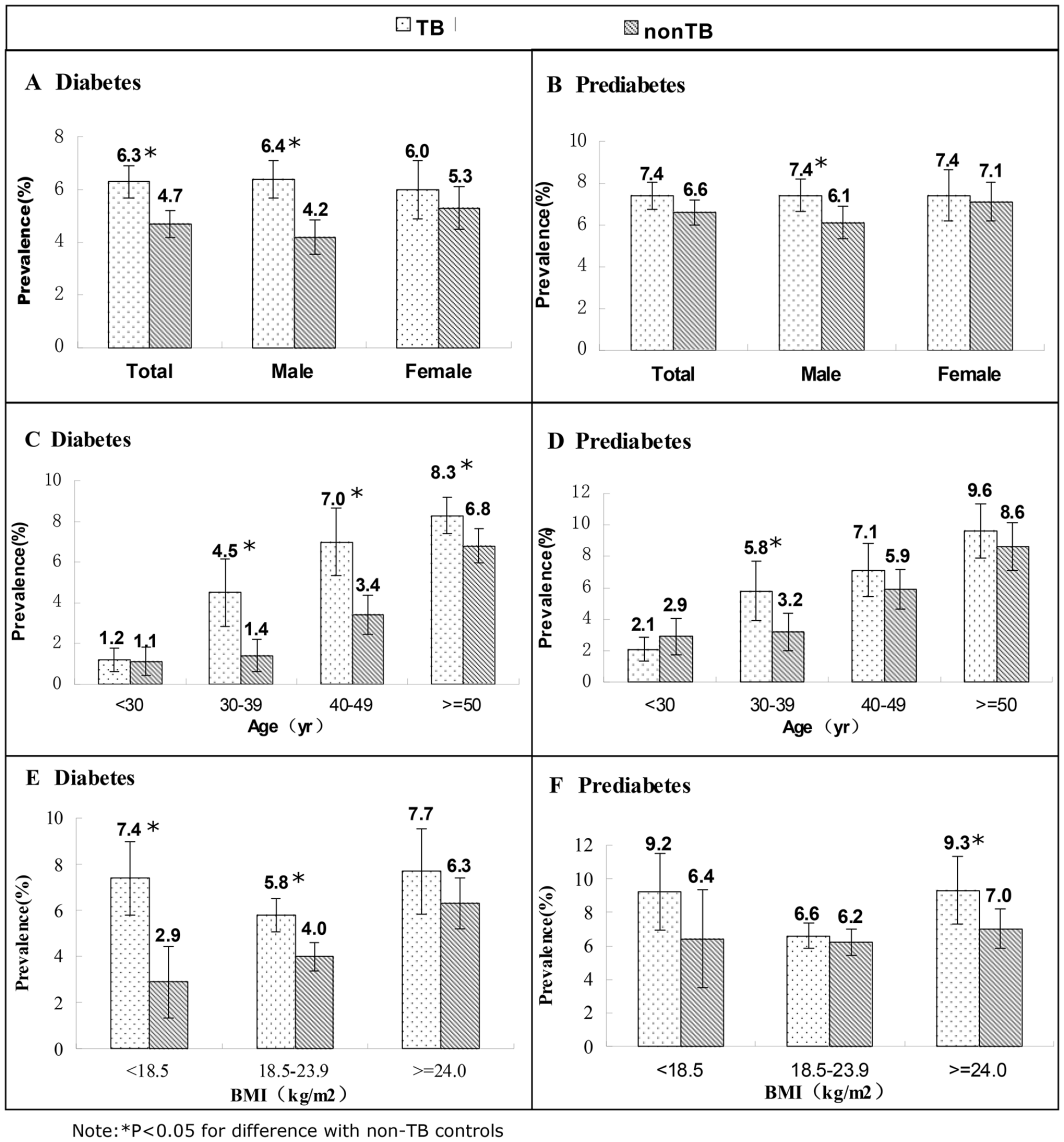
The prevalence of diabetes and prediabetes in TB and non-TB. The overall prevalence of diabetes and prediabetes in TB and non-TB was shown in Figure 2. Also, the sex, age (<30, 30∼39, 40∼49, ≥50) and BMI (<18.5, 18.5∼23.9, ≥24.0) stratified prevalences were shown. The prevalence of diabetes in PTB patients was 6.3%, which was significantly higher than in non-TB controls (4.7%). The prevalence of diabetes increased with increasing age and was significantly higher in the TB than in the non-TB groups except for the age group <30. Among BMI subgroups in TB patients, normal weight patients with a body mass index of 18.5–23.9 had the lowest prevalence of DM (5.8%), while the prevalence in overweight and obese cases (BMI≥24) was the highest. TB: pulmonary tuberculosis patients, non-TB: non tuberculosis controls.

Details of the DM and pre-DM prevalence in TB patients and non-TB controls are shown in Figure 2. Based on FPG, out of 6382 TB patients, 403 (6.3%) had DM; while out of 6675 non-TB controls, 313 (4.7%) had DM. The prevalence of DM in TB patients was significantly higher than in non-TB controls (p<0.05). In males, the prevalence of DM was 52% higher in TB than in non-TB (p<0.05). Taking into account possible confounding factors such as age, sex, BMI, family history of DM, education level, smoking, alcohol consumption, outdoor activity and marital status, we found that PTB patients had 3.17 times higher odds of having DM compared with non-TB control (Table 2).

**Table 2:** Odds Ratio for diabetes and prediabetes by PTB in Linyi, China, 2010–2012.

	Diabetes				Prediabetes			
	Crude OR	p	Adjusted OR*	p	Crude OR	p	Adjusted OR*	p
**overall**	1.38(1.19–1.61)	<0.001	3.17(1.14–8.84)	0.027	1.15(1.01–1.32)	0.041	1.14(0.50–2.57)	0.76
**male**	1.59(1.35–1.94)	<0.001	3.27(1.06–10.06)	0.039	1.25(1.05–1.48)	0.013	1.09(0.46–2.56)	0.84

Adjusted for age, BMI, sex, family history of DM, yearly income, education level, smoking, alcohol consumption, outdoor activity, marital status.

The prevalence of DM increased with increasing age and was significantly higher in the TB groups than in the non-TB groups except for the age subgroup <30. The same tendency existed for pre-DM but the difference was only significant in the age subgroup of 30∼39 (Figure 2).

Among BMI subgroups in TB patients, those with normal BMI (18.5–23.9) had the lowest prevalence of DM (5.8%), while the prevalence of DM in overweight and obese cases (BMI≥24) was the highest (7.7%). The greatest difference (2.6 fold) in DM prevalence between TB and non-TB was observed in the underweight group (p<0.05) (Figure 2).

DM was newly detected in 177 (43.9%) PTB patients and in 136 (43.5%) non-TB controls, respectively. In general, patients with previously diagnosed DM had higher fasting plasma glucose levels than did those with newly-diagnosed DM. 10.3% of previously known DM cases had their blood glucose level tested regularly. The prevalence of pre-DM in TB patients and non-TB controls was 7.4% and 6.6%, respectively, all previously undiagnosed.

### Characteristics of TB Patients with DM or Pre-DM

Table 3 shows the comparison of the study characteristics of PTB patients with Non-DM, Pre-DM, and DM.

**Table 3:** Study characteristics of TB patients by diabetes status in Linyi, China, 2010–2012.

Characteristics	Non-DM	Pre-DM	DM	p value
**N**	5509	470	403	
**Male, n(%)**	3992(72.5)	341(72.6)	298(73.9)	0.813
**Age(years)**	49.29±18.97	57.11±14.72*	57.81±14.23*	<0.001
**BMI(kg/m2)**	20.90±2.72	20.86±2.97	21.09±3.06	0.234
Underweight	866(17.2)	95(21.8)*	77(20.8)*	<0.001
Normal	3504(69.5)	265(60.8)*	231(62.3)*	
Overweight and obesity	674(13.4)	76(17.4)*	63(17.0)*	
**Blood Pressure (mmHg)**				
Systolic	119.53±11.26	119.91±11.77	121.62±13.07*	0.024
Diastolic	77.51±7.70	77.85±6.78	78.61±8.61*	0.017
**Fasting glucose (mmol/l)**	4.94±0.60	6.44±0.26*	10.30±4.81* ^&^	<0.001
**Haemoglobin (g/L)**	131.88±16.90	132.30±18.99	131.54±20.08	0.762
**Triglyceride (mmol/l)**	1.19±1.09	1.23±1.47	1.35±0.74*	0.024
**Total cholesterol (mmol/l)**	4.34±1.01	4.61±1.24*	4.75±1.62*	<0.001
**HDLC (mmol/l)**	1.44±0.59	1.65±0.74*	1.47±0.52&	<0.001
**Type of PTB** * **, n(%)**				
Smear positive cases	2513 (46.8)	237 (55.3)	198 (61.6)*	<0.001
Smear negative cases	2857 (53.2)	192 (44.7)	123 (38.4)	
**Relapse, n(%)**	247 (4.6)	24 (5.6)	28 (8.8)*	<0.001

Note:

p<0.05 compared with non-DM group,

^&^ p<0.05 compared with pre-DM group.

Among PTB cases (6382), 403 patients had DM and 470 had pre-DM. PTB patients with DM and pre-DM were older than the subjects with normoglycemia (57.81, 57.11 vs 49.29, p<0.001). PTB patients with DM had a higher systolic and diastolic blood pressure in comparison with patients with normoglycemia and pre-DM (p = 0.024, 0.017, respectively). Triglyceride and total cholesterol were higher compared with patients with normoglycemia (p = 0.024, <0.001, respectively).

Profile of PTB also showed significant difference. A higher proportion of PTB patients with DM were smear positive (61.6%) compared to those with normoglycaemia (46.8%). A small proportion of patients were categorised as PTB relapse, and patients with DM had the highest relapse rate (p<0.001).

### Odds Ratios for DM and Pre-DM among PTB Patients by Multivariate Risk Assessment

In the multivariate logistic regression models, increasing age, family history of DM, positive sputum smear, cavity on chest X-ray and higher yearly income (≥10000 RMB yuan) were significantly associated with an increased odds of DM in TB patients. Underweight had a borderline significant association with DM compared with normal weight (OR = 1.30, p = 0.082). Frequent outdoor activity was negatively associated with DM odds. Age category of ≥50 years had the highest odds ratio of 13.20 (p<0.001), followed by age category of 40∼ years and family history of DM with OR 9.46, 5.85 respectively. Associations were not observed for sex, education, smoking, alcohol consumption, marital status and involved lung field on chest X-ray (Table 4).

**Table 4:** Multivariate-adjusted odds ratios for diabetes and prediabetes in pulmonary tuberculosis patients.

Significant variables	Diabetes OR (95% CI)	p value	Prediabetes OR(95% CI)	p value
**Age(years)**				
<30(reference)	–	–	–	–
30∼	5.35 (2.47–11.57)	<0.001	3.61 (1.96–6.64)	<0.001
40∼	9.46 (4.61–19.43)	<0.001	4.29 (2.40–7.66)	<0.001
≥50	13.20 (6.71–25.96)	<0.001	5.74 (3.37–9.76)	<0.001
**DM Family history**	5.85 (4.44–7.69)	<0.001	1.58 (1.14–2.20)	0.006
**BMI**				
18.5–23.9 (reference)	–	–	–	–
<18.5	1.30 (0.97–1.76)	0.082	1.38 (1.05–1.80)	0.019
≥24.0	1.08 (0.77–1.53)	0.644	1.44 (1.07–1.93)	0.015
**Positive sputum smear**	1.61(1.08–2.40)	0.021	1.36 (0.96–1.94)	0.085
**Cavity on chest X-ray**	1.66(1.07–2.59)	0.025	0.76(0.46–1.26)	0.285
**Frequent outdoor activity**	0.63 (0.49–0.80)	<0.001	0.72 (0.58–0.89)	0.003
**Yearly income(RMB)**				
<2000 (reference)	–	–	–	–
2000–9999	1.34 (0.94–1.92)	0.106	0.87 (0.65–1.16)	0.337
≥10000	1.65 (1.09–2.50)	0.017	0.95 (0.67–1.34)	0.768

nonsignificant variables: sex, marital status, smoking, alcohol consumption, educational level, involved lung field.

As to having pre-DM, age, family history of DM, overweight, obesity and underweight were positively associated, and frequent outdoor activity was also negatively associated with pre-DM odds.

## Discussion

The present study shows that the prevalence of DM based on FPG values was higher in the PTB (6.3%) patients than in the non-TB (4.7%) controls, and PTB patients had 3.17 times the odds of having DM as non-TB subjects, when adjusted for possible confounding factors.

Our study was designed to discover the prevalence of DM and pre-DM in TB patients and estimate the odds ratios. The strength of this study is its community based setting. To avoid selection bias, we chose community based TB clinics instead of TB hospitals, which means the prevalence and odds ratios can be regarded as representative for the general population. Also, the large sample size allowed us to provide relatively precise estimates of current prevalence of DM in TB. To accurately define DM, we used primary data collected from newly-diagnosed PTB patients and also in a non-TB control group. In other related studies, either the DM prevalence in non-TB was not reported or the prevalence being calculated from secondary data with inherent biases [19], [20]. Only a few reports concerning the prevalence of DM in active TB patients in China are available until now. Zhang Q et al carried out a retrospective analysis in Shanghai pulmonary hospital from 2008–2009 and discovered 9.2% TB patients were complicated by DM [21]. Also from Shanghai, in 1997, Lin S et al reported 4.86% prevalence of DM in TB cases, DM tending to be more prevalent among TB patients in urban area and in older patients [22]. Liang Li et al reported a prevalence of 12.4% in a study based in different parts of China, primarily based on hospital data using FPG [23]. Until now, there were no community based data on the prevalence of DM among sufficiently large samples of TB patients as well as comparative data from non-TB controls from the same community collected in the same time period.

It has been reported that PTB may induce temporary hyperglycemia, which resolves with treatment [14]. Thus, over-diagnosis might take place if tested for glucose prior to initiation of TB treatment. Most of our study subjects were screened for DM 2–3 weeks after the initiation of TB treatment.

We found that the associated factors for having DM were basically the same for PTB patients as for the general population. Increasing age, family history of DM and high income were positive associated factors for DM in PTB patients. For BMI the pattern looks somewhat different. The World Health Organization's (WHO) “standard” definition for underweight, overweight, and obesity by BMI are ≤18.5 kg/m^2^, 25.0 to 29.9 kg/m^2^, and ≥30.0 kg/m^2^, respectively [24]. But in China as in other parts of Asia, a modified criteria is more appropriate to forecast the risk of DM, dyslipidemia and the related diseases [17] and we used this criteria for the cut-off value. The severe underweight and moderate underweight categories only account for 3.4% and 3.7% of the TB patients respectively in our study, so we combined them with mild underweight accounting for totally 17.7% underweight patients in our study. Similarly, obesity only accounts for 0.7% in the PTB cases and we combined it with the overweight subgroup, together accounting for 13.9% overweight and obese PTB cases. Although the average BMI in PTB patients with DM, pre-DM and normoglycemia did not show obvious difference, in logistic regression analysis, underweight (BMI<18.5 kg/m^2^) showed a significant association with pre-DM and borderline significance for DM. This finding was more or less similar to the study carried out in Tanzania [25] that reported severe underweight (BMI<16 kg/m^2^) among male TB patients was associated with DM (OR 2.52, p = 0.004). Underweight is a risk factor for many chronic diseases such as respiratory diseases, osteoporosis, as well as DM [26]. Like in the general population, TB patients with overweight and obesity were at 1.44 fold odds of having pre-DM compared to normal weight patients. But the association was not significant for DM. However, the proportion of overweight and obesity in patients with DM (17.0%) was higher than in patients without DM (13.4%). The association for BMI in the setting of co-morbid DM and TB is complex. While increasing the risk of DM and pre-DM, overweight and obesity is protective against TB disease [27]. Weight loss due to poorly controlled DM and metabolic de-compensation takes away this protection. Presence of TB increases risk of hyperglycaemia through stress but by causing weight loss reduces the risk. These interactions may play out differently in different individuals [28].

The association of positive sputum smear with DM in TB patients was similar to what has been reported earlier [20], [29]. In our study, patients with DM had the highest rate of positive sputum smear, which indicates high risk of infectivity and spread of TB by these patients. Presence of cavities on chest X-rays was significantly associated with co-morbid DM in our study.

Over half of our DM cases (56.1%) were already diagnosed previously. This was lower than nearly all DM patients being aware in the report by Restrepo BI et al [19] and about 75% being aware of DM in the study by Liang Li et al [23]. This difference maybe because our study population was primarily from rural areas where DM awareness and screening are limited. In this context the importance of strengthening monitoring and control of DM becomes relevant. In the present study, only 10.3% of previously known DM cases had their blood glucose level tested regularly. The need for improving DM care services as well as health education about the link between TB and DM cannot be overemphasized, particularly in areas with double burden of the diseases. Our findings show that the opportunities for preventing TB among DM patients should receive greater attention by the health system. DM patients should be aware of their increased risk of active TB. They should be educated to report to TB clinics in time when suspicious TB symptoms occur in order to benefit from having early diagnosis and treatment. Furthermore, underweight persons with DM should be paid high attention given the observed positive association in our study.

Also, updating the educational curriculum of health professionals to increase their awareness of the re-emerging association between TB and DM is urgently required. We discovered that the link of TB and DM was not prioritized in the seven TB clinics that participated in our project, three of which even did not measure the blood glucose level of the TB patients before we started this project. Within the framework of the current project, we implemented health education on the links between TB and DM for TB patients, health care providers and the lay public, the results of which will be reported later.

However, our study also has some limitations. First, since isolated hyperglycemia 2 hours after glucose loading is common among Asian patients with DM [30], we may have underestimated the prevalence of DM because an oral glucose tolerance test (OGTT) was not done for practical reasons. However, the same level of underestimation will apply for the non-TB group and thus does not change the relative odds.

In Shanghai Diabetes Study, 48.6% of patients with newly-diagnosed DM had isolated hyperglycemia 2 hours after glucose loading [31]. So, the actual prevalence rates of DM and pre-DM in our study may have been much higher. This may also explain the difference between our findings and other studies carried out where dual burden of TB and DM also exists. The prevalence rates of DM and pre-DM were 25.3% and 24.5% respectively among TB patients in South India [20].

Another lacuna in our study is that M. tuberculosis (MTB) infection was not confirmed by sputum culture in a substantial number of participants in the clinical settings in our study. However, according to the recent national TB epidemiological survey in China, positive MTB culture is documented in only 26.4% of the active PTB patients [32], which means that nearly three quarter of the active PTB may be missed if we would take the MTB culture result as the gold standard. As with most TB centres, clinical manifestations, sputum smear microscopy and chest radiographs were the central component for PTB diagnosis in our study.

Our study provides further evidence of the links between DM and TB and the evidence that this interaction is playing out even in the rural settings in China where the presumed burden of DM appears low. Our findings indicate the potential benefits of partially integrating TB and DM prevention and control systems, and males may benefit the most given the higher prevalence of TB among them. Given the growing huge burden of DM in China and the high rate of undetected DM, systematic screening of all TB patients for DM through the infrastructure for TB control could serve to improve the early detection of DM, particularly in developing countries. Liang Li et al indicate that such screening has the potential of identifying almost 30,000 new cases of DM each year in China [23]. Follow-up studies are needed to identify the causal mechanism of the link between TB and DM in the future.
